# Diffusion tensor MR neurography reveals altered sciatic and tibial nerve microstructure in chronic dialysis patients

**DOI:** 10.1093/ckj/sfag348

**Published:** 2026-10-01

**Authors:** Melissa Fesselier, Jonas Busecke, Katrin Klein, Clara Raudonat, Georges Sam, Markus Weiler, Sarah Khan-Blouki, Johann M E Jende, Sabine Heiland, Martin Zeier, Martin Bendszus, Christoph M Mooshage

**Affiliations:** Department of Neuroradiology, University Hospital Heidelberg, Heidelberg, Germany; Department of Neuroradiology, University Hospital Heidelberg, Heidelberg, Germany; Department of Nephrology, Heidelberg University Hospital, Heidelberg University, Heidelberg, Germany; Department of Neuroradiology, University Hospital Heidelberg, Heidelberg, Germany; Department of Neurology, University Hospital Heidelberg, Heidelberg, Germany; Department of Neurology, University Hospital Heidelberg, Heidelberg, Germany; Department of Nephrology, Heidelberg University Hospital, Heidelberg University, Heidelberg, Germany; Division of Radiology, German Cancer Research Center (DKFZ), Heidelberg, Germany; Department of Neuroradiology, University Hospital Heidelberg, Heidelberg, Germany; Department of Nephrology, Heidelberg University Hospital, Heidelberg University, Heidelberg, Germany; Department of Neuroradiology, University Hospital Heidelberg, Heidelberg, Germany; Department of Neuroradiology, University Hospital Heidelberg, Heidelberg, Germany

**Keywords:** biomarkers, dialysis, hemodialysis, magnetic resonance imaging, sciatic neuropathy

## Abstract

**Introduction:**

Uremic polyneuropathy is a complication of chronic kidney disease, presenting as a distal symmetric neuropathy mainly attributed to axonal damage. Electrophysiology provides limited insight into microstructural nerve alterations. Magnetic resonance neurography (MRN) may enable assessment of peripheral nerve integrity. This study aimed to quantify sciatic and tibial nerve microstructural changes in chronic dialysis patients and evaluate their clinical relevance.

**Methods:**

Thirty patients on chronic dialysis (DP)—without prediabetes or diabetes—and 30 healthy controls (HCs) underwent MRN, including diffusion tensor imaging (DTI). All MRN datasets of the distal thigh (30/30) were suitable for analysis. Four lower-leg examinations in the patient cohort (26/30) and three in the healthy group (27/30) were excluded because of motion artifacts. Fractional anisotropy (FA) was defined as the primary imaging outcome, while axial diffusivity (AD) and radial diffusivity (RD) were analyzed as secondary metrics. Electrophysiological testing was not available in four patients in the dialysis cohort (26/30). Results were compared between groups and correlated with electrophysiological, clinical, and laboratory findings.

**Results:**

FA of the sciatic (DP 0.44, HC 0.48, *P* < .001) and tibial nerve (DP 0.38, HC 0.43, *P* < .0001) and AD were significantly reduced in dialysis patients, while RD did not differ between groups. Lower FA correlated with electrophysiological measures, neuropathy scores, and was independently associated with group after adjustment for age, BMI, and sex. Subgroup analyses showed lower FA in patients fulfilling electrophysiological criteria for uremic polyneuropathy but no difference according to dialysis modality.

**Discussion:**

The reduction in FA and AD, together with electrophysiological and clinical correlations, is compatible with a predominantly axonal pattern of peripheral nerve involvement in chronic dialysis patients. DTI detected peripheral nerve microstructural alterations associated with chronic dialysis. These findings support further longitudinal and disease control studies to determine the clinical utility of DTI as an imaging biomarker in chronic dialysis patients.

## Graphical Abstract

**Graphical Abstract:**
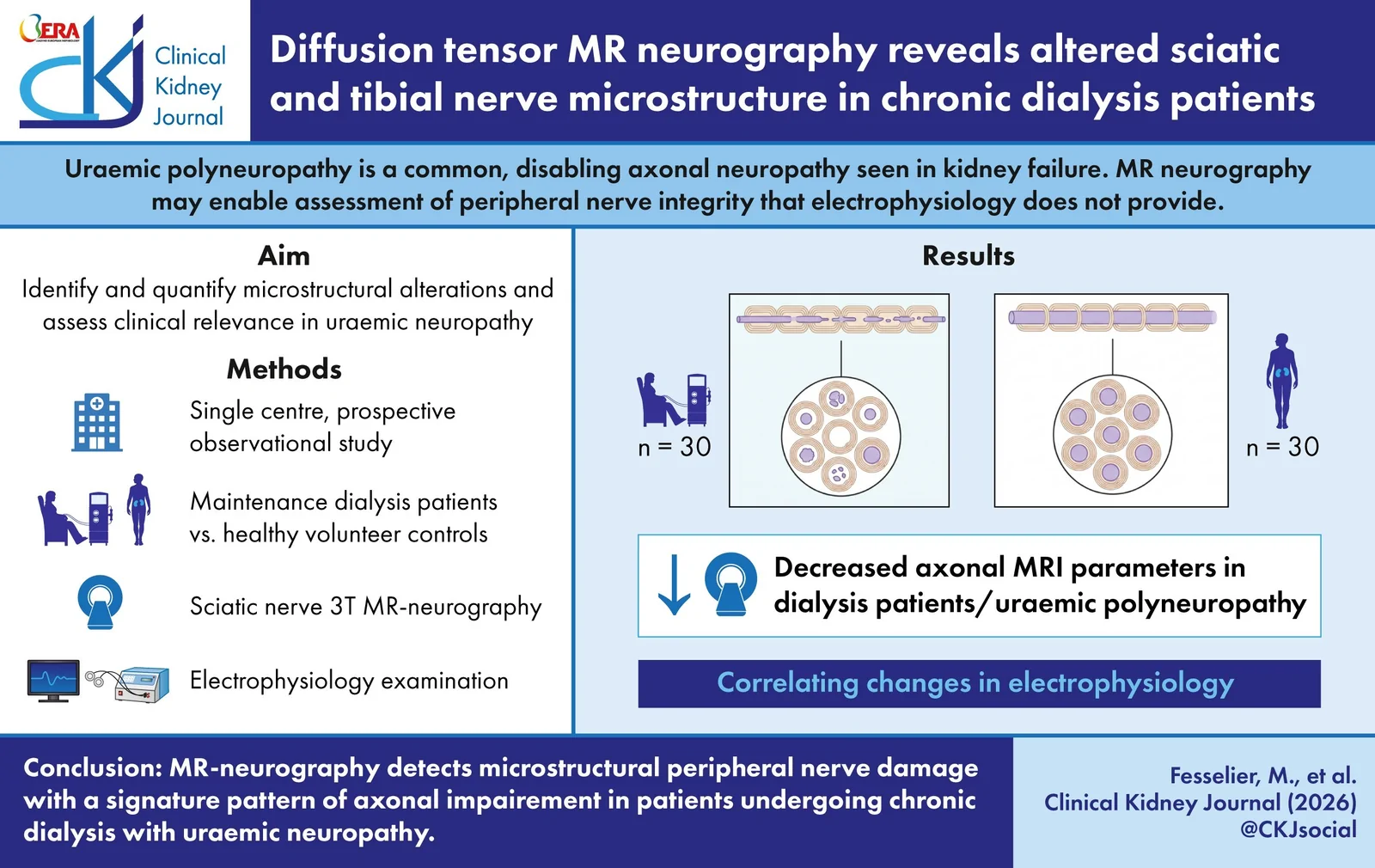

KEY LEARNING POINTS **What was known:**

- Uremic polyneuropathy is a frequent and disabling complication of end-stage kidney disease, usually presenting as a distal symmetric neuropathy and traditionally considered predominantly axonal.
- Clinical examination and electrophysiology remain the diagnostic standard, but they provide limited information on the *in vivo* microstructural nerve alterations underlying uremic polyneuropathy.
- MR neurography with diffusion tensor imaging has shown value in other diffuse neuropathies, but lower-limb microstructural nerve changes in nondiabetic chronic dialysis patients remained poorly characterized.

**This study adds:**

- Chronic dialysis patients without diabetes or other major neuropathy risk factors showed significantly reduced fractional anisotropy and axial diffusivity in the sciatic and tibial nerves compared with healthy controls.
- Preserved radial diffusivity, together with reduced axial diffusivity, supports a predominantly axonal pattern of nerve injury in uremic polyneuropathy.
- Fractional anisotropy correlated with nerve conduction velocities and sensory and motor amplitudes and clinical scores, supporting its role as a noninvasive imaging biomarker of peripheral nerve integrity.

**Potential impact:**

- MR neurography with DTI may complement clinical and electrophysiological assessment by enabling noninvasive detection and characterization of peripheral nerve damage in chronic dialysis patients.
- Quantitative DTI metrics may help distinguish uremic neuropathy from other neuropathy subtypes, particularly diabetic polyneuropathy, when clinical risk factors overlap.
- Earlier and more specific detection of uremic nerve injury could support improved monitoring, risk stratification, and future evaluation of neuroprotective or dialysis-related interventions.

## INTRODUCTION

Peripheral neuropathy (PNP) represents a diverse group of disorders characterized by damage to the peripheral nerves, leading to a range of broad clinical symptoms including sensory deficits, motor weakness, pain, and autonomic dysfunction. Distal and symmetric PNP is the most frequent clinical manifestation. A variety of metabolic and vascular risk factors have been implicated in the development of PNP with diabetes mellitus representing the leading cause of peripheral nerve damage in the western world. Other metabolic contributors include alcohol abuse, obesity, and chronic kidney disease (CKD), often coexisting simultaneously [1, 2]. Uremic polyneuropathy (UPNP) is known to be one of the most debilitating side effects of end-stage kidney disease (ESKD). UPNP is a distal, symmetrical, length-dependent sensorimotor axonal polyneuropathy occurring in advanced CKD, particularly in patients with ESKD. Clinically, it presents with progressive sensory impairment, paresthesia, reduced tendon reflexes and, in advanced stages in muscle weakness. Electrophysiologically, UPNP is characterized predominantly by axonal degeneration with reduced sensory and motor nerve action potential amplitudes, while conduction velocities are usually normal or only mildly decreased. Pathophysiologically, chronic exposure to uremic toxins resulting from impaired renal clearance, together with oxidative stress, chronic inflammation, and metabolic disturbances, is believed to contribute to peripheral nerve injury. As it is often associated with diabetes mellitus, both polyneuropathies may possibly interact and aggravate symptoms. The prevalence of UPNP is reported of up to 60% in dialysis patients; however, most of these studies did not account for potential confounding factors such as diabetes mellitus [3]. Therefore, the validity of these findings for nondiabetic patients is limited.

Liyanage *et al*. [4] estimated a marked increase of dialysis-dependent patients with a projection for 2030 of 4.902 million persons needing dialysis support. Consequently, the incidence of CKD-associated disorders, in particular PNP, will likely increase. Hence, a better understanding of UPNP appears of high importance in these patients.

Concerning the assessment of PNP, a combination of clinical examinations, including neuropathy scores such as the Neuropathy Symptom Score (NSS) or the neuropathy disability score (NDS) and electrophysiological examinations of the peripheral nerves, are considered as the “gold standard” in the assessment of PNP [5, 6]. In recent years, magnetic resonance neurography (MRN) has emerged as a valuable noninvasive imaging technique for the evaluation of peripheral nerve disorders. Beyond depicting nerve morphology, MRN provides quantitative imaging markers that reflect structural nerve alterations. For instance, MRN has previously been applied in diffuse neuropathies such as diabetic PNP and familial amyloid polyneuropathy, allowing new *in vivo* insights into pathophysiological mechanisms and patterns of peripheral nerve damage in these patients [7–9]. Despite some early studies using ultrasound of the median nerve to investigate nerve architecture in pre- and postdialysis patients, little is known about the microstructural nerve alterations in UPNP in dialysis patients, especially affecting the nerves of the lower limb, which are assumed to be impacted first [10]. Although electrophysiological studies remain the diagnostic reference standard and nerve ultrasound allows the assessment of nerve morphology, neither technique directly characterizes peripheral nerve microstructure. MRN, particularly when combined with diffusion tensor imaging (DTI), provides complementary information by visualizing nerve architecture and quantifying tissue microstructural integrity. To the best of our knowledge, studies combining MRN with DTI to assess peripheral nerve microstructure in chronic dialysis patients have not yet been reported. DTI represents a functional MRN technique increasingly used to assess peripheral nerve integrity by measuring diffusion metrics along peripheral nerves. It enables characterization of nerve microstructure and directed nerve diffusion through the main outcome parameter fractional anisotropy (FA) [11, 12].

Therefore, the aim of the present study was to comprehensively characterize sciatic nerve abnormalities in nondiabetic patients undergoing chronic dialysis using DTI MRN, and to investigate their associations with clinical, serological, and electrophysiological parameters.

## MATERIALS AND METHODS

This prospective, single-center observational study was approved by the local institutional review board (S-203/2023) and conducted following the principles outlined in the Declaration of Helsinki. All participants provided written informed consent before participation. Thirty patients with CKD on chronic dialysis (20 on hemodialysis, 10 on peritoneal dialysis; 9 females, 21 males; mean age 59.2 ± 18.8 years; range 28–86) and 30 healthy control (HCs) subjects (20 females, 10 males; mean age 59.4 ± 15.8 years; range 26–88) were included. All participants underwent thorough clinical, serological, and electrophysiological assessments. Patients were prospectively recruited irrespective of the presence or absence of neuropathic symptoms or a previous diagnosis of UPNP. Peripheral polyneuropathy was diagnosed based on nerve conduction studies, which served as the diagnostic reference standard, together with neurological examination. Among the dialysis cohort, 12 patients fulfilled the electrophysiological criteria for peripheral polyneuropathy. Given the presence of ESKD and the exclusion of alternative causes of neuropathy, these cases were considered compatible with UPNP. All participants were recruited through the outpatient clinics of the Heidelberg Kidney Center and underwent MRN of the right thigh and lower leg at 3 Tesla. Since UPNP is generally considered a symmetric, length-dependent polyneuropathy without expected side predominance, MRN and electrophysiological examinations were consistently performed on the right lower extremity to standardize image acquisition and facilitate intersubject comparisons.

Exclusion criteria were as follows: other potential risk factors for the development of neuropathy (e.g. diabetes mellitus, known vitamin B_12_ or folate deficiency documented in the medical record or available laboratory data clinically documented alcohol dependence, malignancy, systemic inflammatory diseases), chronic neurological disease (e.g. multiple sclerosis), or previous exposure to neurotoxic drugs (e.g. chemotherapy), younger than 18 years, pregnancy, any contraindications to MRI, and previous spine surgery. Diabetes mellitus was excluded based on medical history as well as HbA1c measurements and fasting glucose measurements. Available HbA1c values were reviewed for evidence of prediabetes according to current diagnostic criteria (HbA1c <5.7% and fasting glucose <100 mg/dl) [13]. Patients with a known vitamin deficiency documented in the medical history or available laboratory records were excluded. Any evidence of acute or chronic infection was excluded via routine laboratory analysis.

### Clinical and electrophysiological assessments

All participants underwent a detailed clinical history and neurological examination, which included the NDS and NSS. Additionally, neuropathic symptoms were assessed using the EORTC QLQ-CIPN20. Although originally developed and validated for chemotherapy-induced PNP, the questionnaire evaluates generic sensory, motor, and autonomic neuropathic symptoms and was used in this study as a standardized patient-reported outcome measure of neuropathic symptom burden rather than as a diagnostic instrument for UPNP [14]. All electrophysiological studies were performed on the right lower limb. Skin temperature was maintained above 32°C throughout electrophysiological testing according to standard recommendations. Standardized protocols were used and procedures were conducted by a certified examiner, using a Dantec™ Keypoint® Focus EMG device (Natus Medical GmbH, Planegg, Germany) [15]. The nerve conduction study (NCS) protocol included the assessment of nerve conduction velocity (NCV) of the tibial, peroneal, and sural nerves, as well as the sensory nerve action potential (SNAP) of the sural nerve and the compound motor action potential (CMAP) and distal motor latencies (DML) of the tibial and peroneal nerves. Electrodiagnostic criteria for neuropathy were based on normative values and consensus guidelines [16, 17].

### Laboratory testing

Blood samples from dialysis patients were obtained as part of routine clinical care and analyzed by the central laboratory of Heidelberg University Hospital. Routine laboratory assessments included complete blood count, HbA1c, fasting glucose, creatinine, liver enzymes (AST (aspartate aminotransferase), ALT (alanine aminotransferase), GGT (γ-glutamyl transferase)), blood lipids (total cholesterol, triglycerides), thyroid function (TSH), potassium, and parameters of mineral and bone metabolism, including calcium, phosphate, parathyroid hormone (PTH), and vitamin D. Vitamin B_12_ and folic acid levels were available when clinically indicated. Available HbA1c values and fasting glucose were reviewed for evidence of diabetes or prediabetes according to current diagnostic criteria. The presence of relevant metabolic or inflammatory conditions was assessed before study inclusion.

In HCs, laboratory testing was performed prospectively as part of the study protocol and included complete blood count, HbA1c, glucose, creatinine, liver enzymes, lipid profile, TSH, vitamin B_12_, and folic acid. Consequently, not all laboratory parameters assessed in HCs were available for every dialysis patient, as patient laboratory data were obtained from routinely available clinical records. In particular, vitamin B_12_ measurements were available in 9 of 30 dialysis patients. Additionally, vitamin B_12_ and folate measurements were unavailable in two HCs because of sample contamination.

### Image analysis and DTI

High-resolution MRN of the right leg applied the following sequence protocol using a 3 Tesla MRI scanner (Magnetom Prisma^fit^, Siemens Healthineers, Erlangen, Germany):

1. Axial high-resolution T2-weighted (T2w) turbo spin echo 2D sequence with spectral fat saturation of the right mid-thigh with the following parameters: repetition time (TR) 5970 ms, echo time (TE) 55 ms, field of view (FOV) 160 × 160 mm^2^, matrix size 512 × 512, slice thickness 4 mm, interslice gap = 0.35 mm, voxel size 0.3 × 0.3 × 4.0 mm^3^, 3 averages, 24 images.
2. DTI with an axial fat-suppressed, diffusion-weighted two-dimensional echo-planar sequence with the following parameters: TR = 5100 ms; TE = 92.8 ms; b = 0 and 1000s/mm^2^; directions = 20; FOV = 160 × 160 mm^2^; matrix size = 128 × 128; slice thickness = 4 mm; voxel size = 1.3 × 1.3 × 4 mm^3^; no interslice gap, 3 averages, 24 slices, 1512 images.

All MR images were pseudonymized and assessed independently by two physicians with 1 and 6 years of experience in MRN. Image readers were blinded to the clinical, laboratory, and electrophysiological findings during image analysis. Any discrepancies regarding nerve segmentation were resolved by consensus. Subsequently, in a first step, manual segmentation of the tibial portion of the sciatic nerve on the distal right thigh, as well as the tibial nerve at the right proximal lower leg, was performed on the axial, fat-saturated T2w sequence using the free-hand region-of-interest (ROI) tool within the 2D viewer of the DICOM-viewer OsiriX MD (Pixmeo Sarl, Switzerland). ROIs encompassed the entire visible nerve cross-section on each analyzed slice while carefully avoiding surrounding fat and adjacent vessels. Segmentations were accepted only when the nerve borders could be identified with sufficient confidence on the anatomical T2w images. Images with severe motion artifacts or insufficient image quality precluding reliable nerve delineation were excluded from analysis. In a second step, maps for DTI parameters FA, radial diffusivity (RD), and axial diffusivity (AD) were generated using the Osirix MD plugin DTI map with an arbitrary noise threshold of 20. The noise threshold was selected empirically based on visual inspection of DTI parameter maps, similar to previously published MRN protocols, and was applied consistently to all examinations to suppress background noise and exclude voxels with insufficient signal intensity [18]. Finally, the segmented nerve masks (first step) were co-registered with the corresponding FA, RD, and AD maps with manual correction of spatial misalignment. Manual correction was only performed when visually apparent misregistration between anatomical and diffusion maps was present. Correction was performed according to a standardized visual procedure. DTI parameters were averaged (15 slices) across all analyzable slices covering the predefined nerve segment. Thereby, precise assessment of the DTI parameters in the corresponding nerve segments was possible. The applied segmentation and DTI analysis approach is based on previously published peripheral nerve MRN protocols that have demonstrated good reproducibility [19, 20].

Tractography and graphical representations were designed with DSI-studio (https://dsi-studio.labsolver.org) and 3D Slicer (version 5.8.1; https://www.slicer.org), respectively.

### Statistical analysis

Data analysis was performed using GraphPad Prism (version 10.5.0 for Mac OS, GraphPad software, Boston, MA, USA). FA was considered the primary DTI outcome parameter, whereas AD and RD were analyzed as secondary exploratory parameters. Normality was assessed using Shapiro–Wilk tests. Group comparisons were conducted using *t*-tests with Welch’s correction or Mann–Whitney tests depending on Gaussian distribution. Correlation analyses between MRI parameters and demographics, electrophysiology, biological parameters, or clinical measures were conducted using Pearson or Spearman coefficients. Correlation analyses were considered exploratory and were interpreted accordingly. As FA was the primary DTI outcome of the study, the effect of sex on FA was assessed using a robust ANOVA with 20% trimmed means, implemented in Jamovi (version 2.6.45.0; WRS2 package), which provides reliable estimates in the presence of nonnormality or outliers. In addition, to account for potential confounding by sex, age, and BMI, a multivariable ANCOVA was performed with group and sex as fixed factors and age and BMI as covariates. Given the exploratory nature of this pilot study and the limited sample size, no formal correction for multiple testing was applied. Findings from correlation analyses should therefore be interpreted as hypothesis-generating. *P* values ≤ .05 were regarded as statistically significant. Values are displayed as mean ± standard deviation or median and interquartile range (IQR) . Where appropriate, effect estimates are presented together with their 95% confidence intervals.

## RESULTS

From June 2024 to July 2025, a total of 30 patients undergoing chronic dialysis (DP) and 30 HCs were enrolled (Fig. 1.). After MRN acquisition and data extraction, the MRN examinations of the lower leg depicting the tibial nerve of four patients and three HCs were excluded due to excessive artifacts, which precluded reliable tibial nerve segmentation. All excluded participants were male. The four excluded patients were all receiving hemodialysis, and only one had severe neuropathy. No obvious differences in age or BMI were observed between participants with evaluable and nonevaluable tibial nerve datasets. Movement artifacts did not affect distal thigh sequences, allowing accurate analysis of the sciatic nerve in both groups, without population loss for this site. Four patients were lost to follow-up between the MRN and electrophysiological assessments, and no electrophysiological data could be obtained for these individuals.

**Figure 1:**
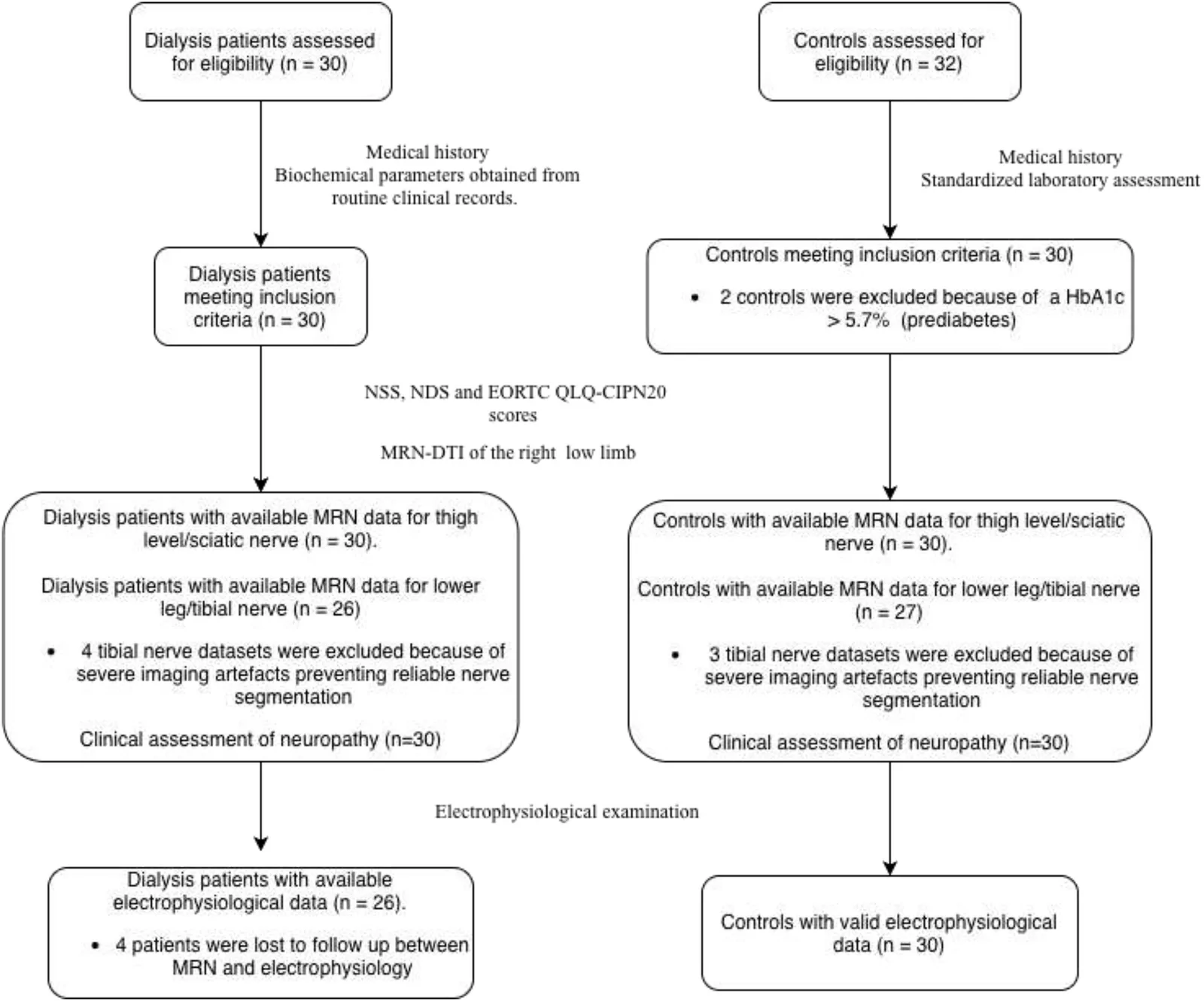
Flowchart of participant recruitment, inclusion, and data availability for MR neurography (MRN), clinical, and electrophysiological assessments.

Demographic, MRN, and electrophysiological characteristics of both DP and HC groups are presented in Table 1. Age and BMI were comparable between both groups, except for an imbalance in sex distribution. Morphologically, nerves of DP appeared with more focal or developed T2w hyperintensities of nerve fascicles and less trackable fibers on nerve DTI tractography (Fig. 2). Electrophysiology showed an overall decrease of NCV, CMAP, and SNAP in patients compared to HCs, while DML was increased in DP cohort (Table 1).

**Figure 2:**
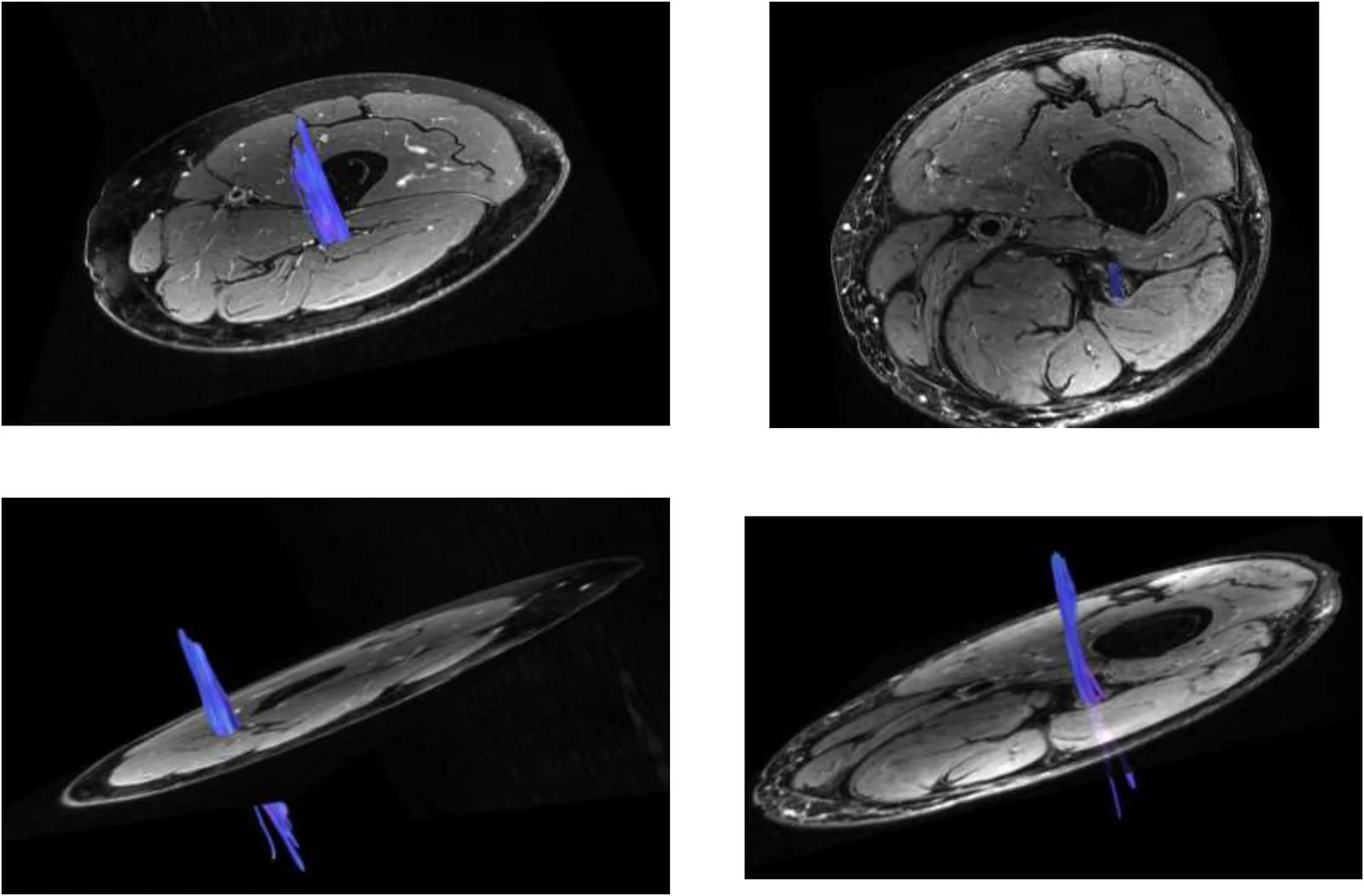
Sciatic nerve diffusion tensor imaging (DTI) tractography of healthy control and patient. Three-dimensional reconstruction of the DTI fibertracking in the sciatic nerve of a healthy control (left) and a uremic polyneuropathy patient (right) at the right thigh level. Overlay of fibertracking on an axial T2-weighted anatomical image. Note the decreased number of trackable fibers between both groups.

**Table 1:** Demographic, electrophysiological, and MRN-DTI characteristics of dialysis patients (DP) and healthy controls (HC).

	Group	*N*	Mean	SD	Minimum	Maximum
Age (years)	DP	30	59.2	18.8	28	86
	HC	30	59.4	15.8	26	88
Sex (M/F)	DP	30	21/9			
	HC	30	10/20			
BMI (kg/m^2^)	DP	30	25.6	4.7	19.5	42.9
	HC	30	24.0	2.9	20.3	31.2
FA_sciatic_	DP	30	0.44	0.06	0.31	0.57
	HC	30	0.48	0.03	0.42	0.56
FA_tibial_	DP	26	0.38	0.05	0.26	0.45
	HC	27	0.43	0.03	0.37	0.49
AD_sciatic_ (10^−3^ mm^2^/s)	DP	30	1.82	0.26	1.11	2.24
	HC	30	2.0	0.17	1.74	2.32
AD_tibial_ (10^−3^ mm^2^/s)	DP	26	2.1	0.19	1.65	2.4
	HC	27	2.21	0.10	1.97	2.39
RD_sciatic_ (10^−3^ mm^2^/s)	DP	30	0.83	0.16	0.49	1.15
	HC	30	0.82	0.10	0.65	1.02
RD_tibial_ (10^−3^ mm^2^/s)	DP	26	1.11	0.14	0.91	1.64
	HC	27	1.10	0.09	0.96	1.35
NCV_peroneal_ (m/s)	DP	26	39.8	8.49	26.4	63.1
	HC	30	48.2	3.55	39.0	56.0
CMAP_peroneal_ (mV)	DP	26	39.8	7.43	28.2	54.7
	HC	30	49.4	3.0	37.3	57.6
NCV_tibial_ (m/s)	DP	26	41.9	7.05	32.8	53.9
	HC	30	50.5	5.23	40.8	60.6
CMAP_tibial_ (mV)	DP	26	9.41	7.56	0.23	29.3
	HC	30	18.6	6.71	5.7	36.6
CMAP_peroneal_ (mV)	DP	26	5.0	3.45	0.11	14.6
	HC	30	8.84	4.07	4.3	20.3
DML_peroneal_ (ms)	DP	26	4.51	0.55	3.61	5.69
	HC	30	4.00	0.55	2.88	4.9
DML_tibial_ (ms)	DP	26	4.07	0.77	2.96	5.5
	HC	30	3.49	0.51	2.37	4.94
SNAP_sural_ (µV)	DP	26	5.38	4.48	0.8	19.1
	HC	30	16.2	6.1	6.1	30.4
NCV_suralis (ms)_	DP	26	41.87	7.05	32.8	53.9
	HC	30	50.47	5.23	40.8	60.6

M = male; F = female; FA = fractional anisotropy; AD = axial diffusivity; RD = radial diffusivity; NCV = nerve conduction velocity, CMAP = compound motor action potential; DML = distal motor latency; SNAP = sensory nerve action potential.

Common main laboratory parameters of patients undergoing dialysis and healthy controls are presented in Table 2.

**Table 2:** Main common laboratory parameters in dialysis patients (DP) and healthy control (HC).

	Group	*N*	Mean	SD	Minimum	Maximum
Creatinine (mg/dl)	DP	30	9.747	2.991	4.440	16.55
	HC	30	0.790	0.164	0.450	1.24
eGFR (ml/min/1.73 m^2^)	DP	30	5.417	2.172	2.700	11.80
	HC	30	89.653	16.045	58.600	131.00
Glucose (mg/dl)	DP	30	86.100	8.739	65	98
	HC	30	94.567	17.210	62	139
HbA1c (%)	DP	30	5.123	0.375	3.900	5.60
	HC	30	5.397	0.228	4.800	5.80
Folate (nmol/l)	DP	26	30.665	16.569	7.000	46.30
	HC	28	30.786	10.556	12.000	45.00
Vitamin B_12_ (pmol/l)	DP	8	472.500	244.501	285	986
	HC	28	388.321	203.499	180	1002
TSH (mU/l)	DP	30	1.750	1.030	0.530	5.88
	HC	30	1.772	1.115	0.516	5.85

eGFR = estimated glomerular filtration rate; TSH = thyroid-stimulating hormone.

The median dialysis vintage was 42 months (IQR, 73.8 months; range, 3–183 months). Twenty patients (66.7%) underwent hemodialysis and 10 (33.3%) peritoneal dialysis. Laboratory parameters are summarized in Table 3. Parathormone and vitamin D measurements were available for 26 of 30 patients, as these tests were not part of routine clinical assessment in four patients. Supplementary biological and dialysis-related characteristics of the dialysis cohort are presented in Table 3.

**Table 3:** Biological and dialysis-related characteristics in dialysis patients.

	*N*	Median	IQR	Minimum	Maximum
Dialysis vintage (months)	30	42.00	73.750	3	183
Potassium (mmol/l)	30	4.93	1.372	3.66	7.46
Parathormone (pmol/l)	26	22.55	29.300	2.00	80.50
Phosphate (mmol/l)	30	1.69	0.385	1.05	3.87
Calcium (mmol/l)	30	2.35	0.120	1.11	2.52
Vitamin D (ng/ml)	26	32.35	17.875	9.40	176.00
CRP (mg/l)	30	2.00	3.60	0.5	23.6
*Dialysis modality*	*n*			%	
Hemodialysis	20	66.7			
Peritoneal dialysis	10	33.3			

CRP = C-reactive protein.

### DTI parameters: comparison between DP and HC

The mean FA value of the sciatic nerve was lower in the DP group (0.44± 0.06) compared to the HC group (0.48± 0.03; *P* = .0006; Fig. 3). Similarly, FA of the tibial nerve was lower in the DP group (median = 0.38, IQR = 0.08) than in HCs (median = 0.43, IQR = 0.03; *U* = 103; *P* < .0001). AD values of the sciatic nerve were also decreased in the DP group (1.82 ±  0.26) compared to HC (2.0 ±  0.17; *P* = .0024). Likewise, AD of the tibial nerve was lower in the DP group (2.1± 0.19) compared to HC (2.21± 0.01; *P* = .0128). In contrast, RD of both nerves did not differ between groups (DP_sciatic_ = 0.83± 0.16, HC_sciatic_ = 0.82± 0.1; *P* = .9; median = 1.112, IQR = 0.119 in DP_tibial_ vs. median = 1.084, IQR = 0.105 in HC_tibial_; *U* = 348, *P* = .96).

**Figure 3:**
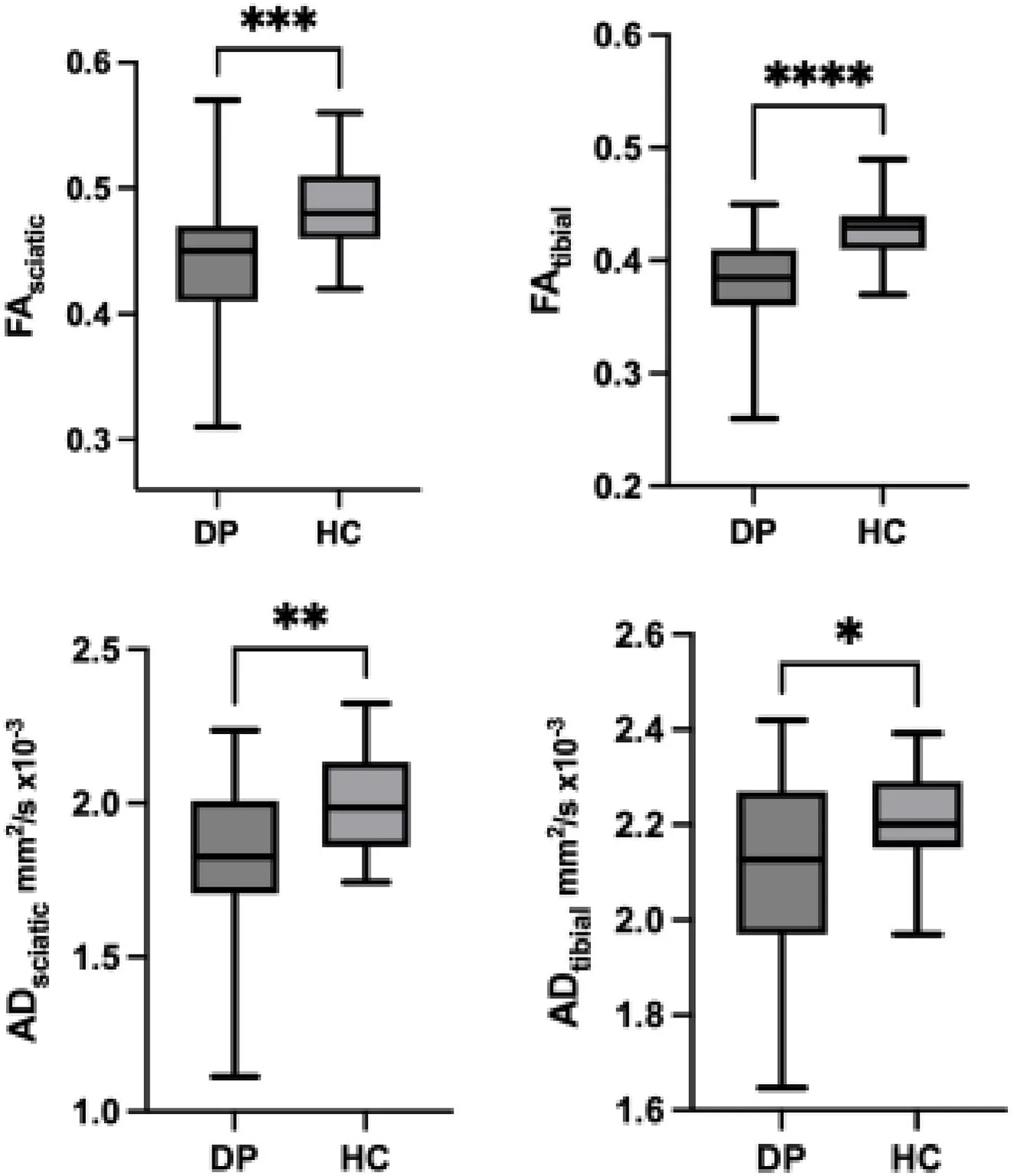
Group comparison of diffusion tensor imaging (DTI) metrics in the sciatic and tibial nerves between dialysis patients (DPs) and healthy controls (HCs). Box plot displays fractional anisotropy (FA) and axial diffusivity (AD) for the sciatic nerve (left panels; DP *n* = 30 and HC *n* = 30) and the tibial nerve (right panels; DP *n* = 26 and HC *n* = 27). Significant reductions of FA and AD were observed in the DP group compared to controls. *P < .05, **P < .01, ***P < .001, ****P < .0001. *P* < .05 was regarded as significant.

**Figure 4.**
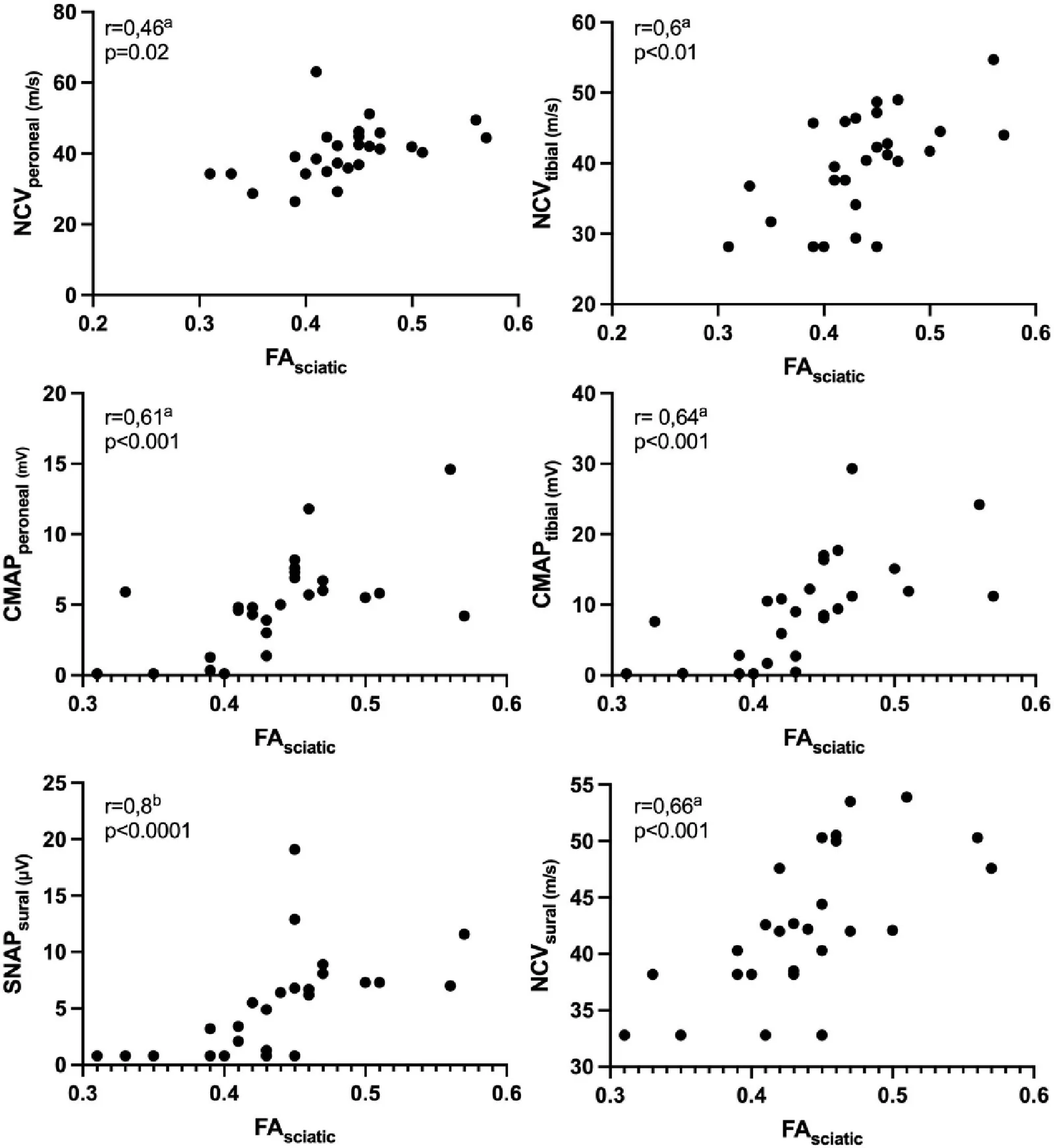
Correlation between fractional anisotropy (FA) of the sciatic nerve and electrophysiological parameters in dialysis patients (UPNP). Scatter plots illustrate the main significant associations between fractional anisotropy (FA) of the sciatic nerve and electrophysiological parameters in dialysis patients (DPs; *n* = 26), including motor nerve conduction velocities (NCV) and compound muscle action potentials (CMAP) of the tibial and peroneal nerves as well as sensory nerve action potentials (SNAP) and velocities (NCV) of the sural nerve. *a* = Pearson; *b* = Spearman. *P* < .05 was regarded as significant.

### Adjustment of FA according to main cofounders

A robust ANOVA with 20% trimmed means was conducted for primary outcome parameter FA to account for sex imbalance between groups. Significant group effects were found for FA_sciatic_ (*Q* = 14.005, *P* = .002), FA_tibial_ (*Q* = 19.173, *P* = .001). The effect of sex was not significant in any model (FA_sciatic_: *Q* = 0.443, *P* = .514; FA_tibial_: *Q* = 1.524, *P* = .238), nor was the group × sex interaction (FA_sciatic_: *Q* = 0.700, *P* = .413; FA_tibial_: *Q* = 0.481, *P* = .5).

After adjustment for age, sex, and BMI, the effect of group on FA in the sciatic nerve remained statistically significant (*F*_(1,54)_ = 9.334, *P* = .003, partial η² = 0.147). Age (*F*_(1,54)_ = 19.047, *P* < .001) and BMI (*F*_(1,54)_ = 7.133, *P* = .010) were independently associated with FA, whereas no significant effect of sex was observed (*F*_(1,54)_ = 1.128, *P* = .293). For the tibial nerve, the effect of group on FA also remained significant after adjustment for sex, age, and BMI (*F*_(1,48)_ = 20.48, *P* < .001, partial η² = 0.299). Age was independently associated with FA (*F*_(1,48)_ = 6.86, *P* = .012), whereas sex (*F*_(1,48)_ = 0.002, *P* = .964) and BMI (*F*_(1,48)_ = 0.891, *P* = .350) were not.

### Correlation: DP MRN parameter FA and electrophysiology

In DP, FA of the sciatic nerve showed positive correlations with multiple electrophysiological parameters (Table 4). These included NCV_peroneal_ (*r* = 0.46, *P* = .02), NCV_tibial_ (*r* = 0.6, *P* < .001), NCV_sural_ (*r* = 0.66, *P* < .001), SNAP_sural_ (*r* = 0.80, *P* < .001), CMAP_tibial_ (*r* = 0.64, *P* < .001), and CMAP_peroneal_ (*r* = 0.61, *P* < .001; Fig. 4). No correlation was observed between FA_sciatic_ and the remaining electrophysiological parameters. In an analogous way in DP, FA of the tibial nerve also positively correlated with NCV_peroneal_ (*r* = 0.62, *P* = .002), NCV_tibial_ (*r* = 0.62, *P* = .002), NCV_sural_ (*r* = 0.63, *P* = .002), SNAP_sural_ (*r* = 0.74, *P* < .001), CMAP_tibial_ (*r* = 0.64, *P* = .001), and CMAP_peroneal_ (*r* = 0.6, *P* = .003), while no significant correlations were found with the remaining electrophysiological parameters.

**Table 4:** Correlation between electrophysiological parameters and MRN-DTI main outcome FA in dialysis patients (DP).

		FA_scaitic_	FA_tibial_
NCV_peroneal_ (m/s)	Pearson’s *r*	0.460*	
	*P*-value	.02	
	95% CI upper	0.752	
	95% CI lower	0.158	
	Spearman’s ρ		0.620**
	*P*-value		.002
	*N*	26	22
CMAP_peroneal_ (mV)	Pearson’s *r*	0.610***	
	*P*-value	<.001	
	95% CI upper	0.814	
	95% CI lower	0.312	
	Spearman’s ρ		0.601**
	*P*-value		.003
	*N*	26	22
DML_peroneal_ (ms)	Pearson’s *r*	0.050	
	*P*-value	.809	
	95% CI upper	0.429	
	95% CI lower	−0.344	
	Spearman’s ρ		−0.156
	*P*-value		.488
	*N*	26	22
NCV_tibial_ (m/s)	Pearson’s *r*	0.597**	
	*P*-value	.001	
	95% CI upper	0.799	
	95% CI lower	0.272	
	Spearman’s ρ		0.617**
	*P*-value		.002
	*N*	26	22
CMAP_tibial_ (mV)	Pearson’s *r*	0.640***	
	*P*-value	<.001	
	95% CI upper	0.823	
	95% CI lower	0.336	
	Spearman’s ρ		0.644**
	*P*-value		.001
	*N*	26	22
DML_tibial_ (ms)	Pearson’s *r*	−0.199	
	*P*-value	.330	
	95% CI upper	0.204	
	95% CI lower	-0.544	
	Spearman’s ρ		−0.300
	*P*-value		.175
	*N*	26	22
NCV_suralis (ms)_	Spearman’s ρ	0.660***	0.627**
	*P*-value	<.001	.002
	*N*	26	22
SNAP_sural_ (µV)	Spearman’s ρ	0.800***	0.737***
	*P*-value	<.001	<.001
	*N*	26	22

NCV = nerve conduction velocity; DML = distal motor latency; CMAP = compound motor action potential; SNAP = sensory nerve action potential.

*P < .05, **P < .01, ***P < .001.

### Clinical phenotype and correlations with main outcome DTI parameter FA in DP

Clinical neuropathy was assessed using the NDS, NSS, and EORTC QLQ-CIPN20 questionnaire. In the dialysis cohort (*n* = 30), the median NDS was 0.0 (IQR 3.5), the median NSS was 4.0 (IQR 6.75), and the median EORTC QLQ-CIPN20 score was 20.0 (IQR 8.0), indicating overall limited clinical neuropathy despite variable symptom severity (Table 5).

**Table 5:** Clinical neuropathy characteristics of the dialysis cohort.

	*N*	Median	IQR	Minimum	Maximum
NDS	30	0.00	3.50	0	10
NSS	30	4.00	6.75	0	10
EORTC QLQ-CIPN20	30	20.00	8.00	20	61

NDS = Neuropathy Disability Score; NSS = Neuropathy Symptom Score; EORTC QLQ-CIPN20 = European Organization for Research and Treatment of Cancer Quality of Life Questionnaire–Chemotherapy-Induced Peripheral Neuropathy 20-Item Scale.

Correlation analyses were performed to investigate the relationship between clinical neuropathy severity and main outcome FA (Table 6). Significant negative correlations were observed between sciatic nerve FA and all three clinical scores. Lower FA values were associated with higher NDS (ρ = −0.530, *P* = .003), higher NSS (ρ = −0.429, *P* = .018), and higher EORTC QLQ-CIPN20 scores (ρ = −0.502, *P* = .005). In contrast, no significant correlations were found between tibial nerve FA and the clinical scores (all *P* > .05).

**Table 6:** Correlations between clinical neuropathy scores and fractional anisotropy values in the dialysis cohort.

		FA_sciatic_	FA_tibial_
NDS	Spearman’s ρ	−0.530**	−0.273
	*P*-value	.003	.177
	*N*	30	26
NSS	Spearman’s ρ	−0.429*	−0.156
	*P*-value	.018	.448
	*N*	30	26
EORTC QLQ-CIPN20	Spearman’s ρ	−0.502**	*-*0.207
	*P*-value	.005	.309
	*N*	30	26

NDS = Neuropathy Disability Score; NSS = Neuropathy Symptom Score; EORTC QLQ-CIPN20 = European Organization for Research and Treatment of Cancer Quality of Life Questionnaire–Chemotherapy-Induced Peripheral Neuropathy 20-Item Scale.

*P < .05, **P < .01, ***P < .001.

### Correlation of main outcome DTI parameter FA with relevant dialysis parameters in dialysis patients

Within the dialysis group, the dialysis vintage was significantly associated with lower FA values of the sciatic nerve (Spearman’s ρ = −0.481, *P* = .007). Although a similar inverse relationship was observed for the tibial nerve, it did not reach statistical significance (Spearman’s ρ = −0.372, *P* = .061; Table 7). No significant correlations were observed between FA values and dialysis-relevant markers such as serum potassium, creatinine, estimated glomerular filtration rate (eGFR), parathyroid hormone, calcium, or phosphate levels (all *P* > .05). A moderate inverse correlation was found between sciatic nerve FA and vitamin D levels (Spearman’s ρ = −0.442, *P* = .025), whereas the association for the tibial nerve did not reach statistical significance (Spearman’s ρ = −0.394, *P* = .070).

**Table 7:** Correlation of fractional anisotropy with biological parameters and dialysis vintage in dialysis patients.

		FA_sciatic_	FA_tibial_
Potassium (mmol/l)	Pearson’s *r*	−0.184	
	*P*-value	.331	
	95% CI upper	0.189	
	95% CI lower	−0.510	
	Spearman’s ρ		−0.227
	*P*-value		.264
	*N*	30	26
Creatinine (mg/dl)	Pearson’s *r*	−0.056	
	*P*-value	.768	
	95% CI upper	0.310	
	95% CI lower	−0.408	
	Spearman’s ρ		0.030
	*P*-value		.885
	*N*	30	26
eGFR (CKD-EPI ml/min/1.73 m^2^)	Spearman’s ρ	0.252	0.095
	*P*-value	.178	.645
	*N*	30	26
Parathormone (pmol/l)	Spearman’s ρ	−0.013	−0.008
	*P*-value	.952	.972
	*N*	26	22
Calcium (mmol/l)	Spearman’s ρ	0.224	0.159
	*P*-value	.235	.437
	*N*	30	26
Vitamin D (ng/ml)	Spearman’s ρ	−0.442*	−0.394
	*P*-value	.025	.070
	*N*	26	22
Phosphate (mmol/l)	Spearman’s ρ	0.063	0.072
	*P*-value	.741	.727
	*N*	30	26
Dialysis vintage (months)	Spearman’s ρ	−0.481**	−0.372
	*P*-value	.007	.061
	*N*	30	26

eGFR = estimated glomerular filtration rate.

*P < .05, **P < .01.

### Exploratory subgroup analysis by dialysis modality

An exploratory subgroup analysis was performed to compare FA values between patients receiving hemodialysis and those receiving peritoneal dialysis. No significant differences were observed between dialysis modalities for either sciatic nerve FA (Mann–Whitney *U* = 97.0, *P* = .914) or tibial nerve FA (Mann–Whitney *U* = 67.5, *P* = .525). Median sciatic nerve FA was 0.446 [IQR 0.0488] in the hemodialysis group and 0.451 [IQR 0.0543] in the peritoneal dialysis group, whereas median tibial nerve FA was 0.380 [IQR 0.0400] and 0.390 [IQR 0.0375], respectively (Table 8).

**Table 8:** Fractional anisotropy values according to dialysis modality in dialysis patients.

	Dialysis modality	*N*	Median	IQR	Minimum	Maximum
FA_sciatic_	Hemodialysis	20	0.446	0.0488	0.312	0.510
	Peritoneal dialysis	10	0.451	0.0543	0.332	0.568
FA_tibial_	Hemodialysis	16	0.380	0.0400	0.290	0.450
	Peritoneal dialysis	10	0.390	0.0375	0.260	0.420

FA = fractional anisotropy.

### Exploratory subgroup analysis according to electrophysiological evidence of uremic polyneuropathy

Among the 26 dialysis patients who underwent electrophysiological assessment, 12 fulfilled the electrophysiological criteria for UPNP, whereas 14 did not (non-UPNP). An exploratory subgroup analysis was performed comparing dialysis patients with electrophysiological evidence of UPNP and those without UPNP (non-UPNP). Patients with UPNP exhibited significantly lower FA values in both the sciatic nerve (median 0.406 [IQR 0.0501] vs. 0.462 [IQR 0.0405], Mann–Whitney *U* = 11.0, *P* < .001) and the tibial nerve (median 0.360 [IQR 0.0900] vs. 0.400 [IQR 0.0300], Mann–Whitney *U* = 14.0, *P* = .003). No significant differences were observed between groups regarding age, BMI, serum creatinine, eGFR, phosphate, parathyroid hormone, calcium, vitamin D, folate, vitamin B_12_, potassium, glucose, or HbA1c (all *P* > .05) (Table 9). Sex distribution did not differ significantly between patients with and without electrophysiological evidence of UPNP (Fisher’s exact test, *P* = .391).

**Table 9:** Biological and DTI characteristics according to uremic polyneuropathy status with uremic PNP patients (UPNP) and patients without uremic PNP (non-UPNP).

	Group	*N*	Median	IQR	Minimum	Maximum
FA_sciatic_	UPNP	12	0.406	0.0501	0.312	0.453
	Non-UPNP	14	0.462	0.0405	0.417	0.568
FA_tibial_	UPNP	9	0.360	0.0900	0.260	0.390
	Non-UPNP	13	0.400	0.0300	0.310	0.450
Glucose (mg/dl)	UPNP	12	88.000	12.0000	67	98
	Non-UPNP	14	86.500	9.7500	65	97
HbA1c (%)	UPNP	12	5.250	0.4000	4.700	5.500
	Non-UPNP	14	5.050	0.2000	3.900	5.600
Phosphate (mmol/l)	UPNP	12	1.640	0.3175	1.050	2.610
	Non-UPNP	14	1.820	0.6300	1.460	3.870
Parathormone (pmol/l)	UPNP	10	35.100	27.3500	2.800	80.500
	Non-UPNP	14	17.850	19.9750	2.000	79.900
Calcium (mmol/l)	UPNP	12	2.305	0.1475	1.150	2.520
	Non-UPNP	14	2.350	0.0650	1.190	2.420
Vitamin D (ng/ml)	UPNP	11	34.400	12.3000	19.000	43.000
	Non-UPNP	14	28.850	16.5250	9.400	176.000
Folate (nmol/l)	UPNP	11	43.000	20.0000	7.000	46.300
	Non-UPNP	14	23.500	33.0000	8.000	45.000
Vitamin B_12_ (pmol/l)	UPNP	3	371	328.0000	330	986
	Non-UPNP	5	337	243.0000	285	654
Potassium (mmol/l)	UPNP	12	4.885	1.4150	3.890	6.200
	Non-UPNP	14	5.215	1.4400	3.660	7.460
Creatinine (mg/dl)	UPNP	12	9.605	2.1725	4.440	16.550
	Non-UPNP	14	9.370	3.6800	6.270	15.440
eGFR (CKD-EPI ml/min/1.73 m^2^)	UPNP	12	5.000	2.0250	2.700	11.800
	Non-UPNP	14	4.850	2.5500	3.000	8.700
Age (years)	UPNP	12	63.500	17.5000	32	86
	Non-UPNP	14	55.000	43.7500	28	86
BMI (kg/m^2^)	UPNP	12	25.937	3.6976	19.493	42.901
	Non-UPNP	14	24.236	4.3186	21.386	30.863
Sex (M/F)	UPNP	12	10/2			
	Non-UPNP	14	9/5			

BMI = body mass index; FA = fractional anisotropy; eGFR = estimated glomerular filtration rate; F = female; M = male.

After review of the individual electrophysiological records, among the 12 patients fulfilling the electrophysiological criteria for PNP, five (41.7%) exhibited a predominantly axonal pattern. Three patients (25.0%) showed a demyelinating component. In two patients (16.7%), the neuropathy was severe with markedly reduced nerve excitability, precluding precise electrophysiological classification. The remaining two patients (16.7%) demonstrated early PNP, although electrophysiological interpretation was limited by challenging recording conditions related to obesity and/or edema.

## DISCUSSION

To our knowledge, this is the first study to assess microstructural alterations of peripheral nerves in patients undergoing chronic dialysis through MRN. The main findings of our study were a reduced FA and AD of the sciatic and tibial nerves in dialysis patients (DP) compared to HCs, while the RD did not differ between both groups. Furthermore, we found positive correlations between the tibial and sciatic FA of DP and nerve conduction velocities (NCV), SNAP of the sural nerve (SNAP) as well as compound muscle action potentials (CMAP). As FA was defined as the primary imaging outcome, additional analyses focused on this metric consistently confirmed its robustness. The reduction in FA remained significant after adjustment for age, BMI, and sex, supporting a disease-related effect beyond demographic confounders. Although age and BMI were independently associated with FA, the group effect remained significant, indicating that the observed alterations cannot be explained solely by demographic or anthropometric differences.

The application of DTI as a functional MRN sequence to evaluate the integrity of peripheral nerves has been described in various conditions, such as chronic inflammatory demyelinating polyneuropathy (CIDP) and other metabolic disorders such as diabetes mellitus [21, 22]. Among DTI metrics, FA is widely regarded as a key parameter for assessing peripheral nerve health, as it reflects the directional coherence of water diffusion within axons and myelin sheaths [23, 24]. However, interpreting FA changes requires context from other metrics and clinical data. In peripheral neuropathies, electrophysiological studies distinguish axonal versus demyelinating damage: reductions in SNAP or CMAP amplitudes reflect a loss of functional axons [25, 26], whereas pronounced slowing of NCV and prolonged distal latencies indicate demyelination [27, 28]. In our cohort, FA showed strong correlations with CMAP and SNAP amplitudes and weaker but significant associations with nerve conduction velocities, indicating that FA reflects overall structural nerve integrity. Moreover, the significant associations between FA and neuropathy-related clinical scores further support that reduced FA reflects clinically meaningful peripheral nerve dysfunction rather than an isolated imaging abnormality. However, because FA is a composite marker and does not discriminate between axonal and myelin pathology, interpretation requires additional evaluation of directional diffusivities [12, 29]. We found that AD was significantly reduced in the dialysis group, which is consistent with AD’s sensitivity to longitudinal axonal fiber integrity. In contrast, RD remained largely unchanged in our patients relative to controls. The absence of a significant increase in RD is compatible with relatively preserved myelin integrity. However, DTI metrics remain indirect imaging biomarkers that are influenced by biological and technical factors and therefore cannot by themselves distinguish axonal from myelin pathology. Taken together with the electrophysiological findings, the observed DTI pattern is compatible with a predominantly axonal phenotype rather than proving a purely axonal neuropathy. This interpretation is further supported by the electrophysiological profile of our patient cohort with UPNP. Most pathological examinations demonstrated a predominantly axonal pattern, whereas a smaller number of patients showed additional demyelinating features. Importantly, none fulfilled established electrodiagnostic criteria for a primary demyelinating neuropathy. Therefore, the imaging findings should be interpreted together with electrophysiology rather than in isolation. In other words, uremic neuropathy as observed via DTI appears to be a predominantly axonal neuropathy. Our findings are supported by previous studies on DTI-MRN in which DTI discriminated the demyelinating nerve pathology in patients with CIDP and Charcot-Marie-Tooth disease type 1 [30, 31].

Classical and contemporary studies demonstrate that accumulation of uremic toxins—including guanidino compounds, beta2-microglobulin, parathyroid hormone fragments, and myoinositol—exerts direct neurotoxic effects on axons, altering excitability and impairing axonal transport [3, 32–34]. Mitochondrial dysfunction and oxidative stress further contribute to distal axonal “dying-back” while Schwann cells and myelin remain relatively preserved until late stages [35, 36]. CKD is also associated with chronic low-grade inflammation, though with a different inflammatory signature from diabetes [37–39]. In addition, a further contributor unique to CKD is ionic imbalance—particularly hyperkalemia and altered calcium–phosphate homeostasis—which can impair axonal membrane excitability more than myelin [40–43]. Interestingly, exploratory analyses demonstrated no meaningful association between FA and most routinely available laboratory parameters, except for vitamin D. This suggests that chronic cumulative uremic exposure rather than isolated biochemical measurements may better reflect the mechanisms underlying peripheral nerve injury. Given the exploratory nature of these analyses, this observation should be interpreted cautiously.

Taken together, the combination of reduced FA and AD, preserved RD, and the electrophysiological findings is compatible with a predominantly axonal phenotype and consistent with the classical description of uremic neuropathy as a primarily axonal disorder [3, 44].

As the microstructural pattern of peripheral nerve damage may differ between uremic and diabetic polyneuropathy [17, 45, 46 ], the stringent exclusion of diabetic and prediabetic individuals and other neuropathy-associated disorders represents a major strength of our study. This rigorous selection minimizes metabolic confounding and increases confidence that the observed DTI alterations are related to uremic neuropathy rather than diabetic metabolic injury. However, whether these DTI features are truly specific to uremic neuropathy cannot be determined from the present study and will require direct comparisons with disease–control cohorts.

Another strength is the use of DTI MRN alongside standard electrophysiology. This multimodal approach allowed us to correlate imaging parameters with functional nerve parameters as well as validated clinical neuropathy scores, lending support to the validity of FA as an indicator of axonal health in peripheral nerves. Exploratory subgroup analyses further strengthened the interpretation of the primary outcome. No significant differences in FA were observed between patients undergoing hemodialysis and peritoneal dialysis, suggesting that peripheral nerve microstructural alterations are primarily related to CKD and cumulative uremic exposure rather than dialysis modality itself. Yet, this hypothesis has to be validated in larger patient cohorts. Conversely, patients fulfilling electrophysiological criteria for UPNP exhibited lower FA values than those without electrophysiological polyneuropathy, providing additional evidence that FA is associated with clinically relevant peripheral nerve involvement consistent with both electrophysiological abnormalities and clinical manifestations of uremic neuropathy. Although these subgroup analyses were exploratory and limited by sample size, together with the electrophysiological and clinical correlations, they provide additional support for FA as the most clinically relevant imaging metric evaluated in the present study and a promising candidate imaging biomarker for uremic PNP requiring prospective validation.

We acknowledge several limitations as well. The sex distribution differed between the patients and healthy cohort; however, statistical adjustment indicated that this imbalance did not significantly influence the results, reinforcing the likelihood that the observed microstructural changes are disease-related rather than of demographic origin. Laboratory analyses were based on routinely available clinical data in patients. Consequently, dialysis adequacy indices (e.g. Kt/V) were not routinely available and could not be included in the exploratory analyses. Vitamin B_12_ was not available in all dialysis patients because laboratory analyses in this group were based on routinely available clinical data rather than systematic study-specific blood sampling. Therefore, subclinical vitamin deficiency cannot be fully excluded as a potential confounder. Although manual correction may introduce observer dependency, identical procedures were applied to all examinations. Although the DTI analysis followed previously published and reproducible protocols, formal inter- and intrarater reliability analyses were not performed in the present study. The exclusion of seven tibial nerve DTI datasets because of motion and imaging artefacts highlights a technical limitation of tibial nerve imaging. Although excluded participants did not appear to differ substantially from those included, apart from being exclusively male, this reduced data availability may limit the generalizability and potential clinical applicability of tibial nerve DTI. Furthermore, DTI metrics should be regarded as indirect markers of peripheral nerve microstructure. Factors such as fascicular architecture, edema, ROI placement, motion artifacts, and hydration status may influence diffusivity measures. Consequently, our findings should be interpreted as supportive rather than definitive evidence of a predominantly axonal phenotype. Taken together, these limitations, including the relatively small sample size and the imbalanced sex distribution, indicate that confirmation of our findings in larger prospective cohorts is warranted before the clinical applicability of DTI can be firmly established.

Furthermore, the sample size may limit the generalizability of our findings. This limited cohort size is partly due to the complex recruitment process, which included stringent exclusion criteria—particularly the exclusion of patients with diabetes or prediabetes—to minimize confounding effects on peripheral nerve integrity. Given the exploratory nature and the cohort size, we did not apply corrections for multiple comparisons.

In summary, this DTI-based MR neurography study demonstrates significant alterations in peripheral nerve microstructural imaging metrics in patients undergoing chronic dialysis. Reduced FA and AD, interpreted together with the predominantly axonal electrophysiological profile, are compatible with a predominantly axonal pattern of nerve injury. Although DTI metrics remain indirect biomarkers that cannot by themselves distinguish axonal from myelin pathology, the consistent associations between FA and electrophysiological parameters, validated clinical neuropathy scores, and exploratory subgroup analyses support FA as the most clinically relevant imaging metric evaluated in this study and a promising candidate imaging biomarker of peripheral nerve involvement in chronic dialysis. These findings provide a rationale for prospective longitudinal studies including disease–control cohorts to determine whether disease-specific DTI signatures can ultimately be established for clinical use.

## Data Availability

Anonymized data will be made available upon reasonable request from any qualified investigator.
